# Recovery of altered neuromuscular junction morphology and muscle function in *mdx* mice after injury

**DOI:** 10.1007/s00018-014-1663-7

**Published:** 2014-06-20

**Authors:** Stephen J. P. Pratt, Sameer B. Shah, Christopher W. Ward, Jaclyn P. Kerr, Joseph P. Stains, Richard M. Lovering

**Affiliations:** 1 Department of Orthopaedics, University of Maryland School of Medicine, 100 Penn St. AHB, Room 540, Baltimore, MD 21201 USA; 2 Department of Physiology, University of Maryland School of Medicine, Baltimore, USA; 3 Department of Orthopaedic Surgery and Bioengineering, University of California, San Diego, USA; 4 University of Maryland School of Nursing, Baltimore, USA

**Keywords:** *mdx*, NMJ, Muscular dystrophy, Eccentric injury, MuSK, Microtubules

## Abstract

Duchenne muscular dystrophy (DMD) is a devastating neuromuscular disease in which weakness, increased susceptibility to muscle injury, and inadequate repair underlie the pathology. While most attention has focused within the muscle fiber, we recently demonstrated significant alterations in the neuromuscular junction (NMJ) morphology and resulting neuromuscular transmission failure (NTF) 24 h after injury in *mdx* mice (murine model for DMD). Here we determine the contribution of NMJ morphology and NTF to the recovery of muscle contractile function post-injury. NMJ morphology and NTF rates were assessed day 0 (immediately after injury) and days 1, 7, 14 and 21 after quadriceps injury. Eccentric injury of the quadriceps resulted in a significant loss of maximal torque in both WT (39 ± 6 %) and *mdx* (76 ± 8 %) with a full recovery in WT by day 7 and in *mdx* by day 21. Post-injury alterations in NMJ morphology and NTF were found only in *mdx*, were limited to days 0 and 1, and were independent of changes in MuSK or AChR expression. Such early changes at the NMJ after injury are consistent with mechanical disruption rather than newly forming NMJs. Furthermore, we show that the dense microtubule network that underlies the NMJ is significantly reduced and disorganized in *mdx* compared to WT. These structural changes at the NMJ may play a role in the increased NMJ disruption and the exaggerated loss of nerve-evoked muscle force seen after injury to dystrophic muscles.

**Electronic supplementary material:**

The online version of this article (doi:10.1007/s00018-014-1663-7) contains supplementary material, which is available to authorized users.

## Introduction

The most common and severe form of muscular dystrophy is Duchenne muscular dystrophy (DMD), a disorder caused by the absence of dystrophin, a structural protein found on the cytoplasmic surface of the sarcolemma. The *mdx* mouse also lacks dystrophin and has been widely used as an animal model of DMD [1]. Dystrophin is part of the dystrophin-associated glycoprotein complex (DGC or DAPC), which connects the internal cytoskeleton of the muscle fiber to the extracellular matrix. The DGC also accumulates at the postsynaptic membrane (aka motor end-plate) of the neuromuscular junction (NMJ), the area of synaptic contact between a motor neuron and its target muscle fiber. The motor end-plate is a specialized area of the sarcolemma that rapidly and consistently responds to release of a neurotransmitter from the overlying nerve terminal. Neuromuscular transmission is normally highly reliable, as each nerve impulse results in the release of more neurotransmitter (acetylcholine) than is required for evoking an action potential in the muscle fiber. This release of ‘surplus’ transmitter and consequent ‘excess’ depolarization of the postsynaptic membrane via acetylcholine receptors (AChRs), often referred to as the ‘safety factor’ [2], ensures that a muscle contraction will occur in response to each nerve impulse, at least in healthy tissue. Proper development and organization at the NMJ is necessary for effective neuromuscular transmission [3, 4], but a number of pathological conditions affecting the distribution of AChRs can lead to a reduction in the safety factor and impairment of neuromuscular transmission [2].

It is now clear that the NMJ in mature skeletal muscle is not a fixed permanent structure [5, 6], but instead possesses a large degree of structural plasticity [7]. The NMJ can display alterations in synaptic organization due to exercise, inactivity, denervation, aging, or crush injury to the nerve/muscle [8–11]. Similarly, the absence of associated proteins can cause changes in structure, and without exception, the NMJ is noticeably disrupted in DMD and *mdx* mice [12–16] and associated deficits in neuromuscular function have been identified [13, 17].

Patients with DMD and *mdx* mice also have increased susceptibility to injury compared to their non-dystrophic counterparts. Over time, this damage/degeneration exceeds the ability to repair/regenerate muscle, leading to irreversible muscle wasting throughout life. In dystrophin-deficient muscle, abnormally high force loss after contraction-induced injury is commonly attributed to structural weakness of the muscle fiber cytoskeleton and changes in signaling [18]. However, we recently reported alterations in NMJ morphology and neuromuscular transmission in *mdx* mice 24 h post injury [19], suggesting that alterations at the NMJ may contribute to the increased injury susceptibility and altered recovery in dystrophic muscle.

The purpose of the current study was to examine changes in morphology and function at the NMJ immediately after injury and throughout recovery. Similar to reports of other hindlimb muscles, we found an increased susceptibility to contraction-induced injury in *mdx* when compared to wild-type (WT) controls. We found alterations in NMJ morphology and neuromuscular transmission only in *mdx* mice immediately and 24 h post-injury. Following injury, we observed a delayed recovery of nerve-evoked muscle force in *mdx* (21 days) compared to wild-type (WT; 7 days). However, despite the severely delayed recovery of contractile function in the *mdx*, the alterations in NMJ morphology and NTF resolved after day 1 post-injury.

Taken together, we conclude that intrinsic alterations at the NMJ in *mdx* mice contribute to the functional deficits seen following muscle injury. We confirm that muscle specific kinase (MuSK), a post-synaptic transmembrane tyrosine kinase important for the clustering of acetylcholine receptors, is significantly reduced in dystrophic muscle. However, neither MuSK nor other constituents of the multi-protein MuSK signaling complex were associated with post-injury alterations in NMJ structure or function. We show that the dense microtubule network that underlies the WT NMJ is significantly reduced in *mdx.* We posit that alterations in microtubule density provide a mechanism for both the early NMJ structural alterations as well as the delay in functional recovery following eccentric injury in the *mdx* mouse.

## Methods

### Animals

We used age-matched male control (WT) and *mdx* (lacking dystrophin) mice from the C57BL/10ScSnJ strain (The Jackson Laboratory, Bar Harbor, ME). A total of 74 mice were used (3 months of age; body weight = 26 ± 0.5 g for WT and 31 ± 2 g for *mdx*, supplemental figure 1). All experimental procedures were approved by the University of Maryland Institutional Animal Care and Use Committee.

### Muscle injury

Quadriceps injury induced by maximal lengthening contractions was performed in vivo as described [20, 21]. With the animal anesthetized under isoflurane and placed in a supine position, the thigh and pelvis were stabilized and the ankle was secured onto a lever arm. The axis of the knee was aligned with the axis of the stepper motor (model T8904, NMB Technologies, Chatsworth, CA) and a torque sensor (QWFK-8 M, Sensotec, Columbus, OH) used to measure torque in Newton-millimeters (Nmm). The femoral nerve was stimulated via subcutaneous needle electrodes (J05 Needle Electrode Needles, 36BTP, Jari Electrode Supply, Gilroy, CA). Proper electrode position was determined by a series of isometric twitches and by observing isolated knee extension in the anesthetized animal. A custom program based on commercial software (Labview version 8.5, National Instruments, Austin, TX) was used to synchronize contractile activation and the onset of forced knee flexion. Injury resulted from 15 forced lengthenings (knee flexion) superimposed onto maximal quadriceps contractions through a 40–100° arc of motion (with full knee extension considered 0°) spaced 1 min apart. This range of motion is similar to one used in human studies [22]. Maximal isometric torque was measured before lengthening contractions and 5 min after the last lengthening contraction, and was used to calculate force deficits. Sham procedures (contractions without lengthening, or passive lengthening without contractions, both with knee immobilized) have been performed [21].

### Muscle recovery

A subset of six animals (3 WT and 3 *mdx*) was used in assessing in vivo functional recovery of injured mouse quadriceps over time. For each animal, maximal isometric torque was measured at five different time points post injury. We report mean percent loss in torque, calculated from pre-injury levels (0 % loss in torque), at each time point for both groups. Time points used were immediately after injury (0 hours, 0 hrs); 24 hours (24 hrs), 7 days (D7), 14 days (D14) and 21 days (D21) post injury. Full recovery in muscle function was considered as percent values *not* significant from 0 % loss in torque (pre-injury torque).

### Assessment of NMJ morphology during recovery

A subset of 15 *mdx* mice were used to assess NMJ morphology at each time point after injury (0, 24 h, D7, D14, D21; *N* = 3 at each time point) and compared them to NMJs from non-injured quadriceps (*N* = 3). Animals were perfusion-fixed through the left ventricle with 4 % paraformaldehyde and the knees were immobilized with a custom designed splint to minimize any differences in muscle length that could occur with knee movement during perfusion. Quadriceps muscles were dissected and stored in fixative until stained with α-Bungarotoxin (BTX) conjugated to Alexa-488 (Molecular Probes B13423, Eugene, OR). Digital images of NMJs from whole mount tissue preparations were obtained with a Zeiss 510 confocal laser-scanning microscope (40× objective for data collection, and 63× objective for representative images) with pinhole set at 1.0 Airy unit. A total of 315 images were collected (non-injured, 68; 0 h, 43; 24 h, 79; D7, 57; D14, 35; D21, 33). Only NMJs in a complete en face view were selected for analysis. This was confirmed using 3-dimensional rotating images of NMJs, reconstructed from confocal Z-stacks (via the projection application in the Zeiss LSM image browser software). A maximum intensity flat plane projection was then made from Z-stacked images in Image-J software (NIH) to account for the depth of the NMJ in measurements. After background was subtracted and noise despeckled, a Gaussian Blur filter with *σ* = 2.00 was applied. Binary images were then generated from which total stained area (TSA) and total stained perimeter (TSP) were quantified. Total area (TA) and total perimeter (TP) were quantified using tracing tools for the complete NMJ endplate. Dispersion index (DI) was calculated as (TSA/TA) * 100, describing NMJ density.

### Assessment of neuromuscular transmission failure

We assessed functional recovery of the NMJ at each time point (0, 24 h, D7, D14, D21 post-injury) in a subset of 15 injured *mdx* mice (*N* = 3 for each time point) and compared them to NMJ function in non-injured quadriceps (*N* = 3). Contractile function of isolated quadriceps muscle was measured as described [20]. The patella tendon was released and secured in a custom-made metal clamp and attached to a load cell (FT03; Grass instruments, West Warwick, RI) using a suture tie (4.0 coated Vicryl). The load cell was mounted to a micromanipulator (Kite Manipulator; World Precision Instruments, Sarasota, FL) so that the quadriceps could be adjusted to resting length. The femur and pelvis were stabilized in a custom-made rig and the femoral nerve was used to stimulate the quadriceps, as described previously [21]. Muscle length was adjusted to obtain maximal isometric twitch force in response to 1-ms monophasic rectangular pulses (L_o_). Tetanic force (achieved by a train duration of 300 ms with 1-ms square pulses at 75 Hz) was recorded, and the signal from the load cell was fed via a DC amplifier (model P122; Grass Instruments) to an analog-to-digital board using acquisition software (PolyVIEW version 16; Grass Instruments). The extent of neuromuscular transmission failure was assessed as previously described [9, 23–25]. The femoral nerve was stimulated (0.2-ms pulses in 80 Hz in 330-ms duration trains every 1 s for 2 min) and every 15 s, a single 80 Hz, 330-ms duration train was applied to the muscle using 7 mm disk tip electrodes (Harvard Apparatus, Inc., Holliston, MA). The relative contribution of neuromuscular transmission failure to muscle fatigue was estimated as: (NF − MF)/(1 − MF), where NF is a percent decrement in force during repetitive nerve stimulation and MF is the percent force decrement during direct muscle stimulation.

### Quantitative RT-PCR

For reverse transcriptase polymerase chain reaction (RT-PCR) detection of NMJ related transcripts, 18 *mdx* quadriceps muscles were harvested and snap frozen from non-injured (*N* = 3) and injured (*N* = 3) animals at different time points (0, 24 h, D7, D14, D21 post-injury). Six non-injured (*N* = 3) and injured (*N* = 3) WT controls were also used to compare to *mdx*. Samples were then homogenized in Trizol reagent (Invitrogen, 15596-026, Carlsbad, CA) and total RNA was extracted according to the manufacturer’s instructions. Subsequently, RNA was reverse transcribed (RevertAid First Strand cDNA Synthesis Kit, Thermo Scientific K1622), and quantitative real time PCR was carried out with an ABI 7300 Sequence Detection System (Applied Biosystems, Foster City, CA) using SYBR green (Maxima SYBR Green/ROX qPCR Master Mix, Thermo Scientific K0222), as described previously [26]. Relative expression was determined by simultaneous comparison to the three “house-keeping” genes; *GAPDH*, *HPRT* and *RPL13*, using the geNorm software (v3.5, Ghent University Hospital, Ghent, BE). Transcripts for the sub-unit acetylcholine receptor alpha 1 (AChRα1) and the protein responsible for AChR clustering [muscle specific kinase (MuSK)] were assessed. The primer sets used for PCR amplification, were: MuSK-F, TGAGAACTGCCCCTTGGAACT and MuSK-R GGGTCTATCAGCAGGCAGCTT; AChRα1-F, CATCGAGGGCGTGAAGTACA and AChRα1-R, ATTCCTCAGCGGCGTTATTG; GAPDH-F, CGTGTTCCTACCCCCAATGT and GAPDH-R, TGTCATCATACTTGGCAGGTTTCT; HPRT-F, AGCAGTACAGCCCCAAAATGG and HPRT-R, AACAAAGTCTGGCCTGTATCCAA; RPL13-F, CGAAACAAGTCCACGGAGTCA and RPL13-R, GAGCTTGGAGCGGTACTCCTT.

### Immunoprecipitation and western blotting

Muscle protein homogenates were prepared from WT and *mdx* whole quadriceps using a lysis buffer made from RIPA (Cell Signaling Technology, 9806S, Danvers, MA), 1 % Sodium Dodecyl Sulfate (Sigma Aldrich, L4390) and 1x Protease Inhibitors (Thermo Scientific, 78441). Samples were pulverized using a Qiagen Tissue Lyser LT (Qiagen, 85600) and then spun twice at 12 K for 10 min at 4 °C. A BCA assay was used to determine protein concentration of the supernatants (BCA Protein Assay Kit, Thermo Scientific, 23227). Samples were spun again at 12 K for 10 min at 4 °C, and then pre-cleared using 50 µL of 50 % slurry Protein G magnetic beads (New England Bio Labs, S1430S) added to 200 μg of protein for 1 h rotating at 4 °C. Following pre-clearing, 12 µL of Guinea Pig anti-MuSK serum [(Nsk-2)-2, gift from Dr. Markus A. Rüegg] was added to samples. Immunocomplexes were allowed to form while rotating overnight at 4 °C. After incubation with 20 µL of 50 % slurry Protein G beads for 1 h at 4 °C, the MuSK-containing immunocomplex was separated using a magnetic separation rack. The beads were washed five times for 5 min each with 0.1 % tween 20/1xPBS to remove non-specific proteins and samples were eluted by adding 20 µL of 2× sample buffer (Life technologies NuPAGE LDS Sample Buffer 4x, NP0007) and 5 % 2-Mercaptoethanol (Sigma Aldrich, M3148). Samples were heated in a dry bath at 95 °C for 5 min. Subsequently, the samples were run on a SDS-PAGE gel and transferred to nitrocellulose. Membranes were blocked for 1 h with 5 % non-fat dry milk in 0.1 % tween 20/1xPBS at room temperature. Primary antibodies against MuSK (rabbit serum 194T/Nsk-2, gift from Dr. Markus A. Rüegg) were applied and rotated overnight at 4 °C. After three washes with 0.1 % tween 20/1xPBS, horseradish peroxidase linked secondary antibody (GE Healthcare Amersham ECL anti-rabbit IgG Horseradish Peroxidase, from donkey NA9340 V) diluted 1:2000 in 5 % NFDM in 0.1 % tween 20/1xPBS was applied at room temperature on a rotating platform for 1 h. After subsequent washing, proteins were then detected using a developing reagent (GE Healthcare Amersham ECL Prime Western Blotting detection Reagent, RPN2232) and imaged on an EpiChem system (UVP BioImaging Systems, Upland, CA).

### Immunolabeling

Animals were perfusion-fixed through the left ventricle with 4 % paraformaldehyde and quadriceps muscles were dissected and snap frozen in liquid nitrogen. Muscle sections were cut longitudinally in a cryostat at 50 µm thick. Sections were immediately post-fixed with 4 % paraformaldehyde for 20 min and then incubated in 3 % BSA in PBS for 1 h. Sections were incubated overnight with primary antibodies against dystrophin (rabbit, Thermo Scientific, RB-9024-P, Fremont, CA) or muscle specific kinase (MuSK), both used at a 1:100 dilution in PBS. After three washes for 10 min each with PBS, samples were incubated in Alexa-488 or Alexa-568 goat anti-rabbit secondary antibodies (Molecular Probes A11034 or A11036, Eugene, OR) at a 1:100 dilution in PBS along with α-Bungarotoxin conjugated to Alexa-488 or Alexa-594 (Molecular Probes B13422 or B13423, Eugene, OR, USA) at a 1:200 dilution in PBS, for 4 h. Samples were washed three times for 10 min each with PBS and mounted in Vectashield (Vector Laboratories, Burlingame, CA). Negative controls were performed using the same protocol but with goat anti-rabbit secondary antibodies and α-Bungarotoxin only. MuSK antibodies were generous gifts from Dr. Steven J. Burden (rabbit 83033) and Dr. Markus A. Rüegg (rabbit serum 194T/Nsk-2).

### Fluorescent labeling and quantification of α-tubulin

The flexor digitorum brevis (FDB) muscles were harvested bilaterally from anesthetized WT and *mdx* mice. Single myofibers were enzymatically isolated in DMEM supplemented with 0.2 % FBS, 1 µl/ml gentamicin, and 4 mg/ml type I collagenase (Sigma, C0130) for 1 h at 37 °C as previously described [27].

Myofibers were plated on extracellular matrix (ECM; Sigma E1270)-coated imaging dishes (Matek, P35G-1.0-14-C), fixed with 4 % paraformaldehyde, permeabilized with 0.1 % Triton X-100 in PBS, blocked in Superblock PBS (Thermo Scientific), stained with BTX-594 (Molecular Probes, B13423) and then labeled with an antibody to α-tubulin conjugated to Alexa Fluor 488 (anti-mouse; Invitrogen 32-2588). Digital images were obtained using a Zeiss 510 confocal laser-scanning microscope. Laser intensity was adjusted on a sample-to-sample basis to maximize the amount of microtubules that are visualized. A 14-image Z-stack was taken at 1 µm intervals to account for total depth of the NMJ, and Image-J (NIH) was used to form a composite image. Background was subtracted uniformly, and the image was transformed into a binary image. The motor-endplate was outlined and the image cropped to isolate microtubule labeling at the NMJ. Total area of pixels at the endplate of myofibers was then quantified in Image J.

### Statistical analysis

Independent variables collected for muscle injury and recovery, neuromuscular transmission failure, NMJ morphology, and qRT-PCR measurements were analyzed by pairwise multiple comparison procedures (SigmaStat, San Rafael, CA). A Holm-Sidak post hoc analysis was performed to determine where significant differences had occurred. For quantification of microtubules, a *T* test was performed. For all statistical analyses significance was set at *p* < 0.05.

## Results

**Fig. 1:**
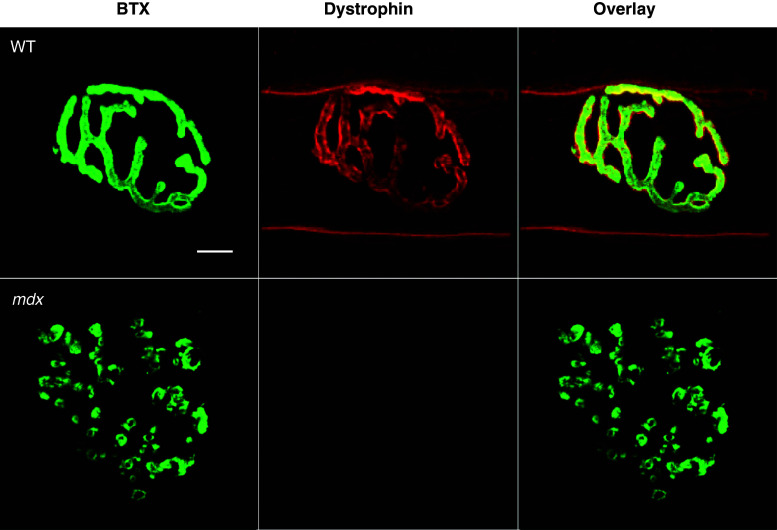
Dystrophin at the neuromuscular junction. Neuromuscular junctions (NMJs) were fluorescently stained with an acetylcholine receptor binding neurotoxin (α-Bungarotoxin, BTX, green), and antibodies against dystropin (*red*). Dystrophin, a stabilizing membrane protein, is present at the NMJ in healthy (wild-type, WT) tissue, but is missing in *mdx* mice. NMJs from *mdx* muscle also show drastic morphological differences when compared with NMJs from WT mice. It has been suggested that the lack of dystrophin is indirectly involved with the fragmented appearance of acetylcholine receptors seen in *mdx* mice. Scale bar equals 10 µm

Motor endplate fragmentation is seen independent of injury in a variety of hindlimb and forelimb muscles of dystrophic mice [13, 16, 28, 29]. Dystrophin is not required for NMJ formation, but is thought to be required for endplate maintenance [16], as the NMJ is significantly altered in *mdx* muscle compared to the NMJ in WT muscle (Fig. 1). Based on a discontinuity index and the number of clusters per NMJ, AChRs in *mdx* mice are more discontinuous and punctate than those in WT mice [19].

**Fig. 2:**
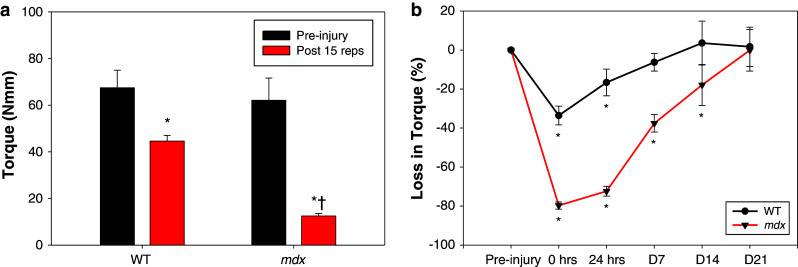
Quadriceps injury and recovery. **a** Pre-injury torque produced from quadriceps muscles in healthy (wild-type, WT) and dystrophic (*mdx*) mice were similar with a maximal torque of 67.5 ± 7.5 and 62.1 ± 9.6 newton millimeters (Nmm), respectively. After 15 forced lengthening contractions, there was a significantly decreased torque production in both control (44.6 ± 2.4 Nmm) and *mdx* (12.5 ± 1.1 Nmm) quadriceps. However, despite an identical injury protocol, *mdx* mice experienced a dramatic 79.7 ± 1.8 % loss in force compared to 33.6 ± 4.8 % in WT mice. All data are presented as mean ± SD, *p* < 0.05. * indicates statistical significance from respective non-injured quadriceps. *†* indicates statistical significance from injured WT quadriceps. **b** The line graph shows the recovery of force production in quadriceps muscles of WT and *mdx* mice over time. Muscle force for WT mice reached pre-injury levels 7 days after injury; however, it took *mdx* mice 3 weeks for full recovery from force deficits. All data are presented as mean ± SD, *p* < 0.05. * indicates statistical significance from pre-injury (0 % loss in torque)

We used a recently described model [19, 21] to injure the quadriceps muscles in WT and *mdx* mice via high force lengthening contractions and performed functional analysis of response to injury in this important locomotory muscle. Before injury, the quadriceps in the WT and *mdx* mice showed a similar mean peak torque of 67.5 ± 7.5 and 62.1 ± 9.6 Nmm, respectively (Fig. 2a). Immediately after injury (0 h), there was a significant loss of maximal torque in both WT and *mdx* mice following an equal number of lengthening contractions. However, the *mdx* quadriceps were significantly more susceptible to 15 lengthening contractions, resulting in a 79.7 ± 1.8 % loss of torque compared to a 33.6 ± 4.8 % loss of torque in WT muscles (Fig. 2a and supplemental figure 2). This increased loss in torque is consistent with data from studies of ankle and respiratory muscle comparing the WT and *mdx* response to injury [30–32]. Recovery from injury in the *mdx* mice was also delayed compared to WT; the WT muscles recovered in 7 days (6.3 ± 4.5 % loss, not significant from pre-injury) whereas the *mdx* muscle took 3 weeks (0.1 ± 10.7 % loss, not significant from pre-injury) to return to pre-injury torque levels (Fig. 2b). To the best of our knowledge, this is the first report comparing the recovery pattern in the quadriceps muscles of healthy and dystrophic mice after lengthening contractions.

**Fig. 3:**
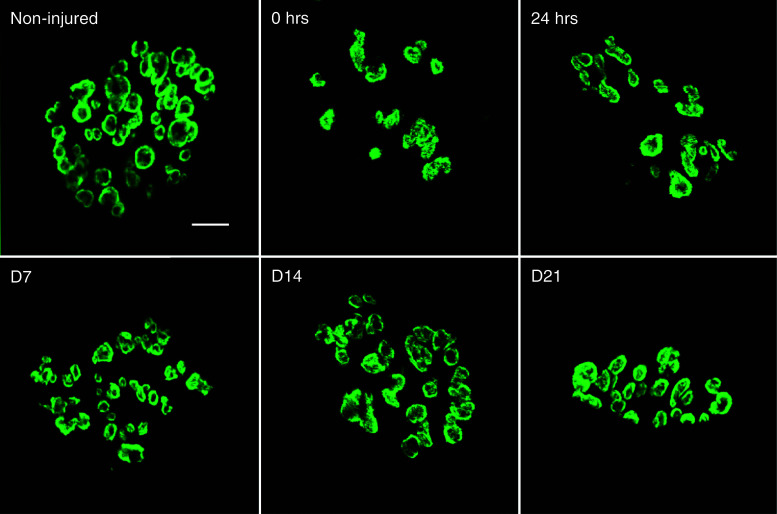
Changes in neuromuscular junction morphology after injury and during recovery. Representative images of neuromuscular junctions (NMJs) from quadriceps muscles of dystrophic (*mdx*) mice were stained with α-Bungarotoxin (BTX) conjugated to Alexa-488 and were imaged using confocal microscopy. A total of 315 Z-stacked images (Non-injured, 68; 0 h, 43; 24 h, 79; D7, 57; D14, 35; D21, 33) were analyzed and quantified using the Dispersion Index (DI, see Table 1). NMJs from non-injured muscle show a non-continuous, punctate pattern of acetylcholine receptor clusters. Immediately (0 h) and 24 h after injury, NMJ morphology is significantly altered, showing a more disperse (smaller DI) acetylcholine receptor morphology. However, NMJ dispersion was not significantly different from non-injured at day 7, day 14 and day 21. Scale bar equals 10 µm

WT NMJs (Fig. 1) show a typical continuous aggregate of AChRs and after injury display no significant change in morphology [19]. Having described NMJ 24 h after injury in *mdx* mice [19], we now show that NMJ disruption occurs in *mdx* muscles immediately after injury (Fig. 3). These changes in morphology were quantified by means of a dispersion index (DI), which was significantly reduced only at two of the selected time points after injury; immediately (0 h) and 24 h after injury (Table 1). The differences indicate a transient reduction in acetylcholine receptor density due to injury that does not persist throughout the recovery period.

**Table 1:** Morphological characteristics of the neuromuscular junction during recovery

	Non-injured	0 h	24 h	Day 7	Day 14	Day 21
TSA	488.1 ± 165.3	587.0 ± 215.3	474.3 ± 220.5	695.9 ± 290.5*	668.1 ± 256.5*	632.4 ± 345.0
TSP	333.8 ± 119.3	299.8 ± 85.3	336.5 ± 99.4	310.4 ± 139.3	323.8 ± 114.8	290.9 ± 116.6
TATP	1,150.8 ± 479.6	1,662.8 ± 534.0*	1,309.9 ± 581.8	1,534.8 ± 697.6*	1,606.6 ± 652.4*	1,549.3 ± 751.5*
TP	142.3 ± 31.1	159.3 ± 30.6	149.6 ± 32.2	150.5 ± 34.0	156.5 ± 33.5	151.0 ± 37.9
DI	45.2 ± 9.6	36.9 ± 10.9*	37.6 ± 8.8*	46.9 ± 8.5	42.8 ± 7.2	41.8 ± 9.8

Data are derived from dystrophic (*mdx*) mouse quadriceps muscles before and after injury

TSA total stained area, *TSP* total stained perimeter, *TA* total area, *TP* total perimeter, *DI* dispersion index

DI was calculated as (TSA/TA) * 100 and describes density of acetylcholine receptors. All data are presented as mean ±SD, *p* < 0.05. *, indicates statistical significance from non-injured

**Fig. 4:**
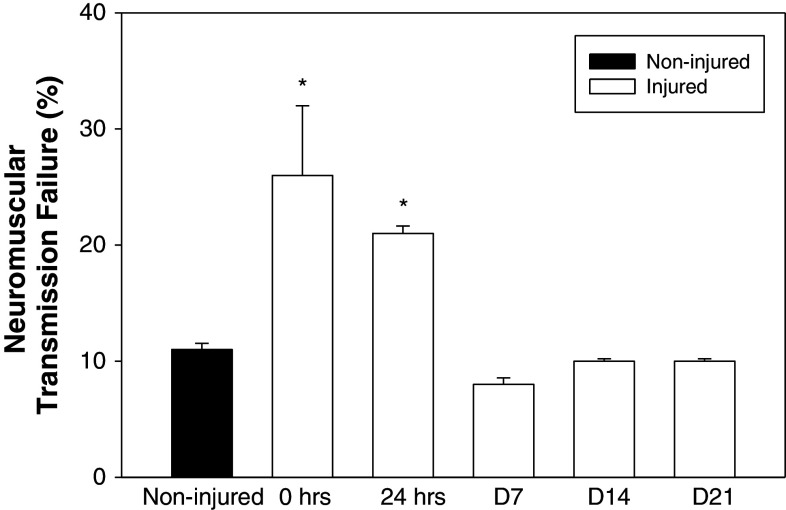
Recovery of neuromuscular function. Neuromuscular function was assessed using the neuromuscular transmission failure (NTF) assay in quadriceps muscles of dystrophic (*mdx*) mice. Baseline NTF rate of neuromuscular junctions (NMJs) from non-injured tissue was 11 ± 0.5 %. NMJs assessed in injured quadriceps immediately (0 h) and 24 h after injury showed a significantly higher NTF rate of 26 ± 6.0 and 21 ± 0.6 %, respectively. NMJs from quadriceps 7, 14 and 21 days after injury had a failure rate that was not significantly different from that of non-injured tissue (8 ± 0.6, 10 ± 0.2 and 10 ± 0.3 %, respectively). All data are presented as mean ±SD, *p* < 0.05. * indicates statistical significance from non-injured

To determine if there were functional changes in the NMJ that paralleled the morphological changes at the NMJ, we estimated the extent of neuromuscular transmission failure during repetitive nerve stimulation (Fig. 4). NMJs in *mdx* showed significant increases in transmission failure immediately after injury and at 24 h (26 ± 6 and 21 ± 1 %, respectively). Interestingly, neuromuscular transmission was no different than in uninjured *mdx* NMJs at all later time points (Fig. 4). This change in neurotransmission parallels the rapid restoration of pre-injury morphology (Fig. 3; Table 1), but does not parallel the continuing whole muscle functional deficits at later time points.

**Fig. 5:**
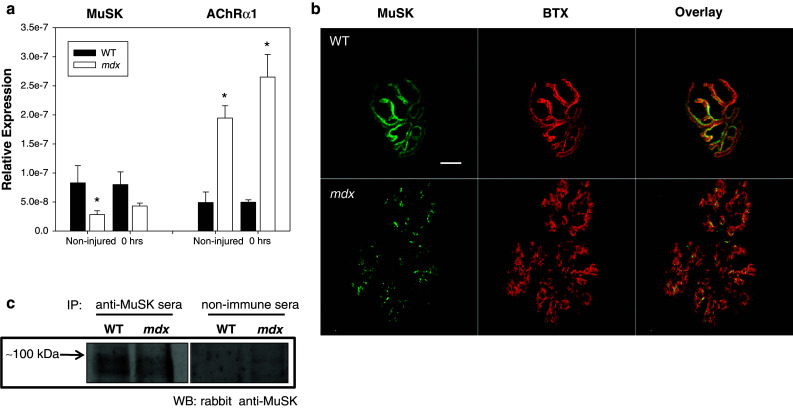
Presence of muscle specific kinase (MuSK) in healthy (WT) and dystrophic (*mdx*) muscle. **a** Transcripts for acetylcholine receptors (AChRs) and muscle specific kinase (MuSK) were quantified using qRT-PCR. Relative expression of MuSK was decreased in *mdx* quadriceps muscles when compared to WT. MuSK, which is important for proper clustering of acetylcholine receptors, may also contribute to the abnormal NMJ morphology seen in *mdx* mice. Interestingly, AChR transcripts were also increased. Expression was unchanged for both AChRs and MuSK following injury (see supplemental for later time points). All data are presented as mean ±SD, *p* < 0.05. * indicates statistical significance from WT. **b** Uninjured neuromuscular junctions (NMJs) were stained with α-Bungarotoxin (BTX, *red*) and labeled with antibodies against muscle specific kinase (MuSK, *green*). NMJs in *mdx* mice show a decrease in MuSK labeling, compared with NMJs from WT mice. Scale bar equals 10 µm. **c** MuSK was immunoprecipitated from extracts of uninjured quadriceps muscle from WT or dystrophic mice, then immunoblotted with anti-MuSK antibodies. Ctrl: immunoprecipitate from muscle probed with a non-immune serum. Equal loading and transfer was confirmed by Ponceau S staining (not shown)

Muscle specific kinase (MuSK) is a transmembrane tyrosine kinase crucial for forming and maintaining the neuromuscular junction and activation of the MuSK complex drives AChR clustering [33, 34]. We previously assessed transcripts for the multi-protein MuSK signaling complex (Agrin, LRP4, Dok7, MuSK and Rapsyn) in WT and *mdx* mice and reported changes only in MuSK [19]. Here, the reduction of MuSK in *mdx* muscle was confirmed both by immunofluorescent labeling (Fig. 5b) and immunoprecipitation of MuSK (Fig. 5c). We also assessed MuSK transcripts from injured *mdx* quadriceps at each time point after injury. The data show that levels of MuSK mRNA, although reduced in *mdx* muscle, were not further altered by injury (Fig. 5a and supplemental figure 3). In addition, we examined mRNA of the AChRs and found a marked increase in AChR transcripts in *mdx* muscle, but no appreciable changes in AChR transcript levels as a result of injury (Fig. 5a and supplemental figure 3).

**Fig. 6:**
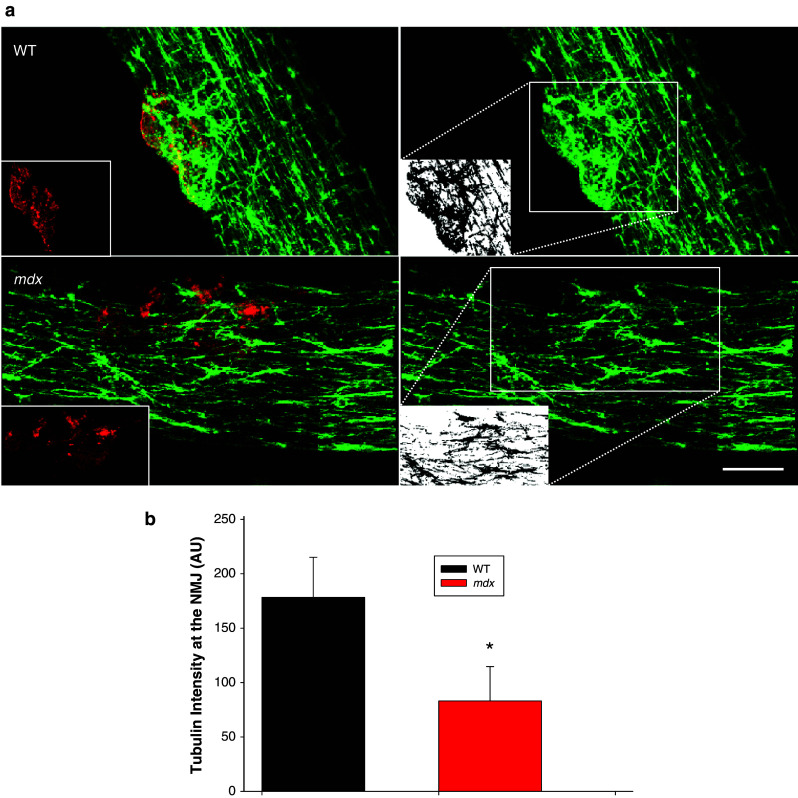
Microtubule structure at the NMJ. To study microtubule architecture underlying the NMJ, intact myofibers from the flexor digitorum brevis muscle (FDB) of wild type (WT) and dystrophic (*mdx*) mice were isolated and plated. **a** The NMJ was identified with α-Bungarotoxin (BTX, red inset) and the associated microtubule structures (green) were examined by labeling of α-tubulin. Labeling shows disorganization of the normal microtubular latticed structure in the *mdx* myofiber, as described by others. Interestingly, the microtubule network density was also significantly decreased at the NMJ in *mdx* muscle. White inset panel shows binarization at the region of interest (*dotted box*). **b** Quantification was performed on binary images of α-tubulin immunohistochemistry (*n* = 2 animals, 5 fibers per genotype; *p < 0.05).  Scale bar equals 10 µm

Since changes in the multi-protein MuSK signaling complex were not associated with post-injury alterations in NMJ structure or function in *mdx* muscles, we hypothesized that deficits in the cytoskeletal network may increase the susceptibility of the NMJ to physical disruption. Alterations in microtubule network density can have a profound impact on contractility in *mdx* muscle [35, 36]. Here we show that the NMJ in the *mdx* contains a significant decrease in microtubule density compared to WT (Fig. 6a, b).

## Discussion

The genetic basis for DMD has been determined [37–39], but the mechanisms responsible for the decrease in muscle specific force and increased susceptibility to injury are still being clarified. The increased force loss after injury is purportedly due to structural weakness of the muscle fiber cytoskeleton or changes in signaling secondary to the loss of dystrophin [18]. We have previously provided evidence that the NMJ, already altered in *mdx* muscle compared to WT muscle, is further perturbed by in vivo eccentric injury [19]. It is unlikely that any one finding can account for the totality of functional changes in damaged muscle; however, normal NMJ structure and function are prerequisites for muscle function in vivo and the NMJ damage seen only in dystrophic muscles provides an additional explanation for the characteristic reduction in specific force and increased susceptibility to damage.

Susceptibility to injury of *mdx* skeletal muscle is well described and recovery from injury, although less studied, is seemingly not delayed. In fact, it has been reported that recovery from eccentric injury to the ankle dorsiflexor muscles is accelerated in *mdx* mice [40, 41]. Using a controlled eccentric injury, we show here that *mdx* quadriceps muscles take much longer to recover (21 days) than WT quadriceps muscles (7 days). There are many possible reasons that our current results conflict with earlier findings from other groups, the most probable being differences in the injury protocols utilized. Another plausible explanation could be the different muscle groups tested. Proximal muscles are affected earlier and to a greater extent in patients with DMD [42, 43], and a similar pattern of increased damage in more proximal muscles has been documented in *mdx* mice [44].

Prior to injury, *mdx* mice present with altered NMJ morphology compared to WT. Muscle specific kinase (MuSK), important for the clustering of acetylcholine receptors (AChRs), is significantly reduced in dystrophic muscle. We confirm at the mRNA and protein levels that MuSK is reduced in *mdx* quadriceps muscles, but not further altered by eccentric injury. Nonetheless, it is plausible that the reduction in MuSK seen in dystrophic muscle contributes to the altered morphology of the NMJs. Reduction of MuSK may also limit agrin-based signaling, which is important for microtubule capture and stability at the NMJ [45].

Recent work has described a novel signaling pathway in which signaling through MuSK triggers downstream signals that capture microtubules at synaptic AChR clusters [45]. Microtubules, components of the cell cytoskeleton with several functions including structural maintenance, play an important role in the pathogenesis of muscular dystrophy [35, 36]. Microtubules bind dystrophin and the normal latticed network of microtubules is disrupted in *mdx* muscle [35]. Our histological examination of the NMJ revealed that microtubule network density was significantly decreased in *mdx* muscle compared to WT muscle. Therefore, the lack of dystrophin and reduction in MuSK signaling results in a reduction in microtubule network that may contribute to the disrupted NMJ structure at rest.

Structure is clearly a major determinant of function in biology, especially in muscle. In the same way that the development of force relies on the controlled overlap of actin and myosin, the apposition of the nerve terminal and the underlying motor end-plate is likely a major determinant of NMJ function. Since MuSK has such a defined role in organization of the motor end-plate [33, 46], our findings showing a reduction in MuSK suggest that it is a viable therapeutic target for DMD.

Alterations in AChRs in dystrophic muscle are well described [47–49]. Here we show further disruption immediately after injury (Fig. 3), suggesting the fragmentation is due to mechanical perturbation and not solely representative of AChR turnover or muscle regeneration. This is consistent with observed mechanical perturbation of NMJ following mechanical loading of either the muscle or nerve [50, 51]. Degeneration and regeneration of the underlying muscle fibers is purportedly responsible for changes in NMJ morphology in aging muscle [52], but several studies suggest that in dystrophic muscle, the changes seen in NMJ morphology are independent of degeneration and regeneration [13, 53]. Moreover, since turnover of AChRs has been reported on a timeline of days [54, 55], it is improbable that a complete biological reorganization of the NMJ and its AChRs occurs immediately after injury.

In healthy WT muscle, the injury protocol used here results in a significant loss of force (~40 %) without corresponding changes in NMJ structure or loss of neurotransmission [19]. Interestingly, for *mdx* mice, by the time NMJ morphology and function “recover” (Day 7), the torque deficit (~40 %) is similar to that of WT muscle immediately after injury. We postulate that disruption of the NMJ, which is limited to early time points, could contribute to the increased force loss seen early after injury in *mdx* muscle.

It was recently reported that transmission of the action potential along the sarcolemma is altered in *mdx* mice after eccentric injury [56]. In that paper, the authors point out that “…there is no evidence for immediate mechanical disruption of the NMJ during eccentric contractions which would abruptly (<1 h) impact strength development”. The current work indicates that the NMJ is indeed disrupted immediately after injury. This work focused on the proximal thigh (quadriceps) muscles, whereas the excellent work by Call et al. was performed in the more distal leg (ankle dorsiflexors) muscles. Given the heterogeneity in the onset and severity of pathology among muscle groups, noted earlier, caution is needed in directly comparing the two studies.

Studies with a number of cell types indicate that mechanical properties of cells are affected by microtubules [57]. Disruption of microtubules results in a rapid increase in the amount of force transferred across the cell surface [58] and may act as a tension reducer and resist cell stretch [59, 60]. A possible role for microtubules in absorbing cell tension, along with the finding that the microtubule network at the NMJ in *mdx* mice is significantly reduced, may contribute to the susceptibility of the NMJ to damage in dystrophic mice.

The cell cytoskeleton resists mechanical loads and is responsible for a cell’s ability to resist shape distortion, but it also provides tracks for the movement of organelles critical cellular functions [61]. Thus, cells that respond to mechanical force with changes in cell shape, and consequently cytoskeletal structure, can have changes in the function of the nearby organelles [62]. It has been proposed that mitochondria move to the sarcolemma at regions of damage along microtubules [35, 63] and that disruption of the microtubule structure may affect how mitochondria respond to injury [35]. We show that the microtubule network at the NMJ in *mdx* mice is significantly reduced, which suggests that such mechanisms are altered and opens an array of questions in regards to mitochondria density/function, excessive reactive oxygen species (ROS) in this region, and other potential changes in the underlying cytoskeletal structure.

In summary, our findings in the quadriceps muscles are similar to previous findings that show increased susceptibility to injury in *mdx* mice, but differ in terms of the speed of recovery. We have also shown that the NMJs in *mdx* muscle are additionally perturbed by forceful eccentric contractions and that these changes occur early and resolve before full muscle function is returned. Because changes in NMJ morphology and function are found so soon after injury, it is unlikely that changes seen in the NMJ are due to nascent, regenerating fibers or some other reorganization of NMJ structure. The data here also show that MuSK is decreased in *mdx* quadriceps muscles, but the question of whether increasing MuSK in dystrophic muscle would rescue the NMJ and improve the whole-muscle phenotype is unknown.

### Electronic supplementary material

Below is the link to the electronic supplementary material.

Supplementary material 1 (TIFF 80 kb)

Supplementary material 2 (TIFF 138 kb)

Supplementary material 3 (TIFF 116 kb)
