# Supplementary figures and images for: Recovery of altered neuromuscular junction morphology and muscle function in mdx mice after injury
Source: Cell Mol Life Sci. 2014 Jun 20;72(1):153–64. doi: 10.1007/s00018-014-1663-7 (PMC4282693; doi:10.1007/s00018-014-1663-7)

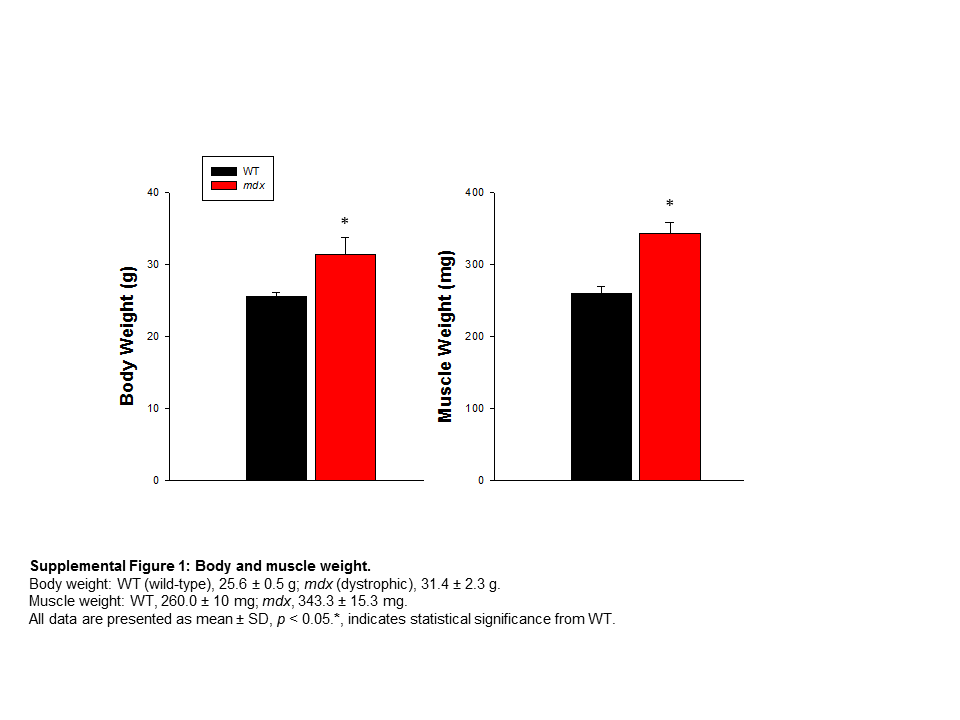

Supplement: Supplementary file 1 — Supplementary material 1 (TIFF 80 kb) [file 18_2014_1663_MOESM1_ESM.tif]

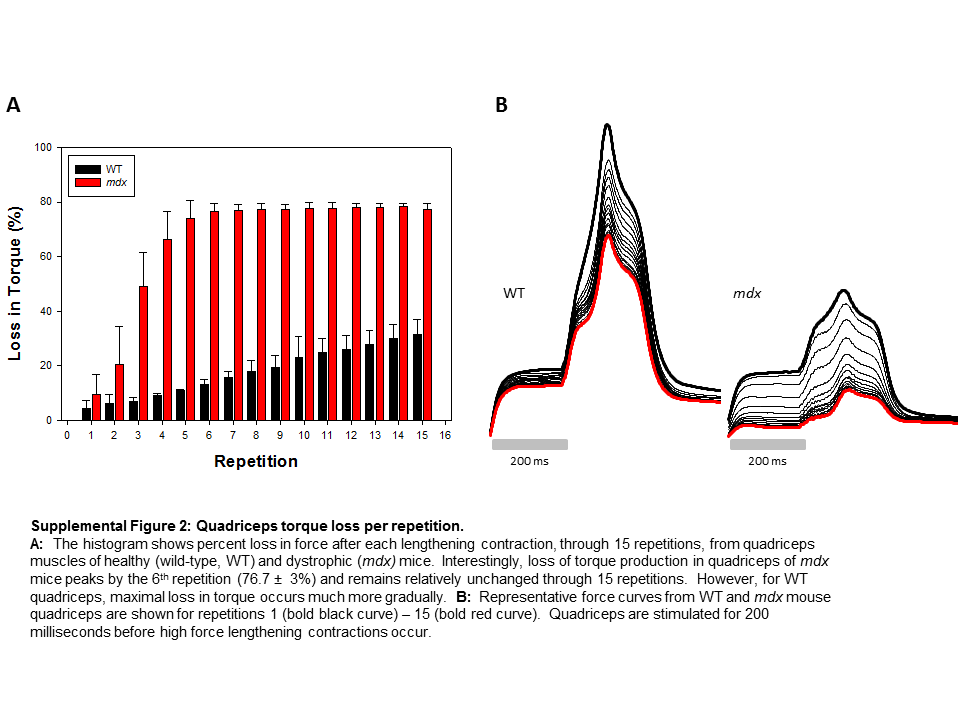

Supplement: Supplementary file 2 — Supplementary material 2 (TIFF 138 kb) [file 18_2014_1663_MOESM2_ESM.tif]

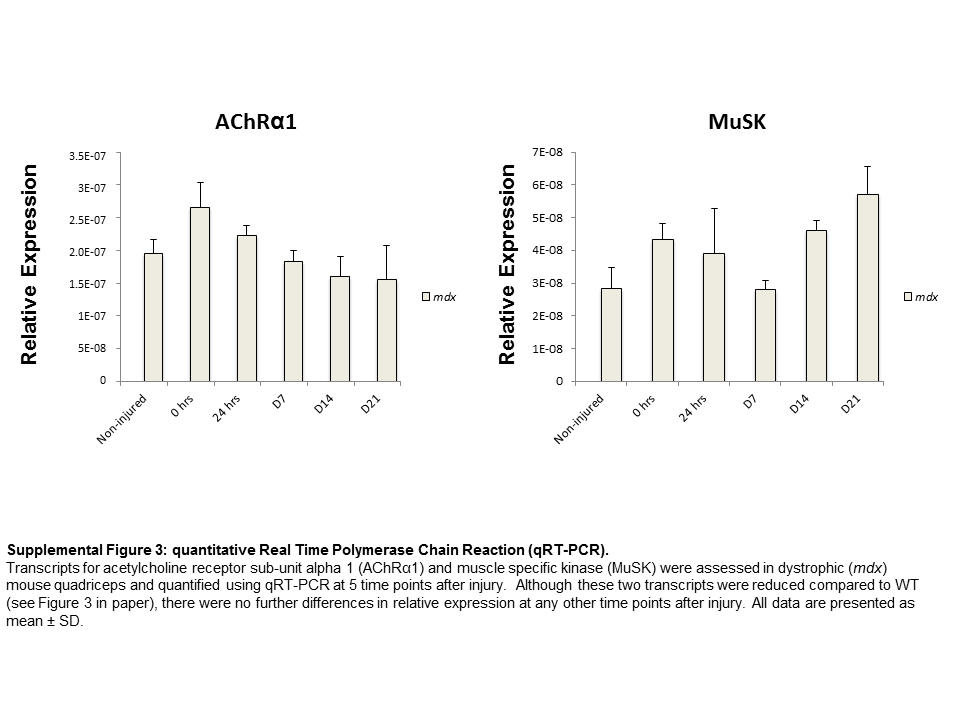

Supplement: Supplementary file 3 — Supplementary material 3 (TIFF 116 kb) [file 18_2014_1663_MOESM3_ESM.tif]
